# Appropriateness and Pattern of Antibiotic Prescription in Pediatric Patients at Adigart General Hospital, Tigray, Ethiopia

**DOI:** 10.1155/2021/6640892

**Published:** 2021-04-10

**Authors:** Adane Yehualaw, Chernet Taferre, Abere Tilahun Bantie, Desalegn Getnet Demsie

**Affiliations:** ^1^Bahir Dar University, Department of Pharmaceutics, Bahir Dar, Ethiopia; ^2^Adigrat University, Department of Anesthesia, Adigrat, Ethiopia; ^3^Adigrat University, Department of Pharmacy, Adigrat, Ethiopia

## Abstract

**Background:**

Inappropriate and unnecessary use of antibiotics can increase morbidity, mortality, medical expenses or patient cost, and microbial antibiotic resistance. However, in developing countries like Ethiopia, information regarding appropriateness of antibiotic prescribing pattern to guide improvement strategies is scant.

**Objective:**

The aim of this study was to assess appropriateness and pattern of antibiotic prescription in pediatric patients at pediatric ward of Adigrat General Hospital.

**Methods:**

Hospital-based retrospective cross-sectional study was conducted to assess the antibiotic prescribing pattern in pediatric inpatient and outpatient ward of Adigrat General Hospital from December 1, 2018 to April 30, 2019. Data was collected by using structured data collection checklist, and the systematic random sampling technique was employed to enroll the required sample size during the study period. Appropriateness of drug use in pediatrics was evaluated using Ethiopian Standard Treatment guideline and WHO pediatric guideline.

**Result:**

A total of 692 pediatric patients' medical charts were reviewed. The median age of patients on antibiotics was 3.26 years (IQR: 2-4). Majority (49.13%) of the patients were hospitalized for 5-9 days. SCAP (195), tonsillitis (114), and cellulitis (99) were most frequently encountered pediatric diseases. Penicillins (37.86%) followed by cephalosporins (31.79%) antibiotics were the most prescribed antibiotics in pediatric wards. This study also showed that ceftriaxone and ceftriaxone+amoxicillin were the most frequently used single and combination antibiotics, respectively. The prescribing practices were not stick to WHO core indicators and standards. Inappropriate prescription of antibiotics was observed in 28.3% of patients. Advanced age of children, children aged between 6 to 10 years (AOR = 3.225; CI = 1.080 − 9.630; *P* = .036) and 11-18 years (AOR = 18.691; CI = 5.156 − 67.756; *P* = .000), was the independent determinant of inappropriate drug use.

**Conclusion:**

Inappropriate antibiotic prescribing was encountered in 28.3% of children. The rate of generic prescription was not in line with WHO recommendation. Advanced age of children was the independent factor for inappropriate use of antibiotics.

## 1. Introduction

Infants and children represent a large part of the population in developing countries. Pediatric group populations are commonly affected by various infectious diseases [1–3].

Antimicrobial agents have been frequently prescribed to children to treat and prevent different infectious diseases in the community and outpatient settings [4–7]. Their use is rational (appropriate, proper, correct) when patients receive for the appropriate indication (right medication), in doses that meet their own individual requirements, for the right duration, at the right route, and at the affordable cost for the right patient [8, 9]. Irrational (inappropriate, improper, incorrect) use of medicines is when one or more of these conditions are not met [10]. Worldwide, it is estimated that over half of all medicines are prescribed, dispensed, or sold inappropriately [11]. However, their irrational use leads to a number of consequences in term of cost, drug interactions, hospital stay, and bacterial resistance, notably penicillin-resistant Streptococcus pneumonia [3, 12–14]. The emergence of antimicrobial resistance causes ever increasing need for new drugs, contributing to the rising cost of medical care, inappropriate and over use of medicines, wastage of resources, and result in significant patient harm in terms of poor patient outcomes and adverse drug reactions [15]. Other studies also demonstrated that inappropriate antibiotic use can result in super infections due to antibiotic-resistant bacteria, opportunistic fungi, unnecessary health care expenditure, treatment failures, and/or adverse drug effects [3, 16].

Irrational antimicrobial use remains to be a major challenge in developing countries. For instance, in Ethiopia, previous literatures indicated inappropriate use of antimicrobials in some hospitals [17–19]. Basically, studies done in Ethiopia are limited in number and do not indicate current status of appropriateness of antibiotic use, and there was not previous or current study done in the study area.

Thus, the aim of this study was to assess appropriateness and prescribing pattern of antibiotics at pediatric wards of Adigrat General Hospital.

## 2. Materials and Methods

### 2.1. Study Area and Period

The study was conducted at Adigrat General Hospital, Adigrat, Tigray, Ethiopia, from December 1, 2018 to April 30, 2019. Adigrat city is located 1903 km away from Addis Ababa. The city is part of the eastern zone of Tigray bordered with Eritrea. Presently, Adigrat General Hospital is serving as a teaching hospital and provides emergency, inpatient, and outpatient services for more than 1.2 million people. The hospital has four wards (internal medicine, pediatrics gynecology and obstetrics, and surgical ward) with around 240 beds.

### 2.2. Study Design

A retrospective cross-sectional study was conducted. Data were collected using a pretested check list. World Health Organization Guideline for Pediatric Illness [20] and Ethiopia Standard Treatment Guideline for General Hospitals [21] were used to assess the rational use and prescribing pattern of antibiotics. Appropriateness was measured according to a recommendation of use in terms of indication, dose, frequency, and duration. Core drug prescribing indicators such as percentage of generic prescription, injection use, and prescription from National Essential Drug List were assessed using WHO and Food, Medicine, and Healthcare Administration and Control Authority of Federal Ministry of Health Care Administration and Control Authority (FMHACA) standards [22–24].

### 2.3. Source and Study Population

The source population was all pediatric patient admitted to pediatric ward of Adigrat Genral Hospital, and the study population was all patients admitted to AGH between July 2013 and August 2018.

### 2.4. Sample Size Determination and Sampling Procedure

A single population proportion formula was used to estimate the minimum sample size required for the study. Since there is no study which shows antibiotic prescription pattern in pediatric patient in the study area, proportion (*p*) = 50% was used, assuming*d* (sampling error) = 5% and using 95% confidence level, where *n* is the sample size, *z* is the statistic for 95% level of confidence, *d* is the precision/margin of error/, *p* is the estimate of the population, and *q* is the 1 and –*p* is the 0.5.

(1) $$\begin{array}{c} n=\frac{Z^{2}\left(1-P\right)P}{d^{2}}=\frac{1.96^{2}\left(1-0.5\right)0.5}{0.5^{2}}=\frac{1.96^{2}\left(0.5\right)0.5}{0.05^{2}}=384. \end{array}$$

However, WHO recommends a minimum sample size of 600 for drug-related surveys on secondary sources [25]. Hence, the final sample size was 692. List of all medical records during the study period was considered as sampling frame, then using unique medical chart numbers as a reference.

### 2.5. Data Collection Procedure

Three nurses and 3 druggists were trained about the data collection process and were familiar with information included in the data abstraction format. Besides, two data collection supervisors were oriented on the data collection process and how to perform follow-up of data collectors. Then, data from 692 medical records were collected using pretested, structured data collection tool that contains sociodemographic characteristics, diagnosis, and treatment related information. In this case, data collectors used a unique medical card identification number to identify and collect data from the selected medical records. Patients admitted with bacterial infections and received antibiotics were included in the study. Patients using antibiotic for prophylaxis purpose were excluded from the study.

### 2.6. Data Quality Control

Before the actual data collection, pretesting was done on five percent of the total sample size in different health institutions. To ensure quality of the data, pretesting of the questionnaire was undertaken in 5 percent of the sample size in similar setups before the actual data collection took place. Data was checked for completeness, accuracy, and clarity at the end of every day of data collection.

### 2.7. Data Processing and Analysis

Data were entered in to Epi-info version 7 and exported to SPSS version 21 for data cleaning and analysis. Sociodemogrphic characteristics of patients, clinical characteristics, and treatment-related information were described in terms of frequency and proportions using descriptive statistics. Univariate and multivariate logistic regression analyses were employed to come up with factors associated with inappropriate use of antibiotics. Bivariate analysis was done to know the association between independent variable and the outcome variable. The multivariate logistic regression model was used to identify the independent determinants of appropriateness of antibiotic prescribing after selecting variables that meet crude odds ratio significance value, i.e., *P* < 0.2 in bivariate analysis. Finally, based on adjusted odds ratios at 95% confidence interval and *P* value <0.05, the variables with significant predictors were identified.

## 3. Results

In our study, a total of 692 pediatric patient medical records with antibiotic drugs prescribed were evaluated. Majority of the participants were males (50.60%). Regarding the age category, 41.62% of the participants were in the age range of 1-5 years, while the least number of participants was below the age of <1 month. 49.13% of the participants were hospitalized for 5-9 days, whereas the second highest rate of hospitalization was reported in the age range of 1-4 years (37.86%) (Table 1).

**Figure 1:**
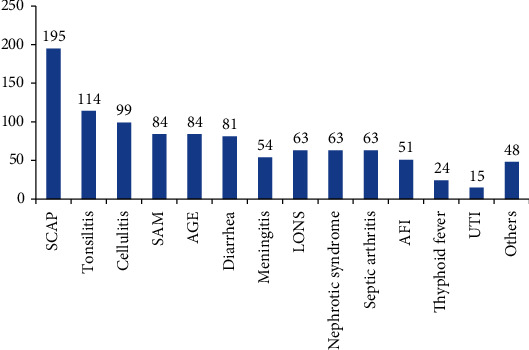
Pattern of infection distribution. SCAP: severe community acquired pneumonia.

The major reasons (conditions) for antibiotic use in pediatric use were SCAP (195), tonsillitis (114), and cellulitis (99) (Figure 1).

Others were AGN, meningitis, typhoid fever, GI Sepsis, AFI, conjunctivtis, H.pylori, and mixed infections.

With regard to antibiotic use, ceftriaxone (45.91%) was the most commonly prescribed drug. Metronidazole was used in 1.36% of prescriptions administered and was given in combination in 7.24% with another antibacterial for systemic use, namely, cloxacillin (Table 2).

From the 718 combination antibiotic prescriptions, ceftriaxone with amoxicillin (200) and ampicillin with gentamicin (195) was more widely prescribed antibiotics, while ceftriaxone with cloxacillin [10] and ampicillin with ciprofloxacin [10] was the least commonly used antibiotic combination in the study area (Table 3).

**Figure 2:**
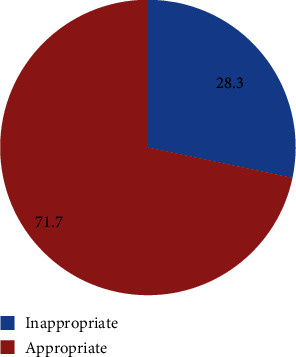
Appropriateness of antibiotic prescription among pediatric patients.

Higher proportion of participants (18.21) encountered inappropriate use of antibiotics due to high or low doses followed by inappropriate duration (10.12) and indication (3.18) (Table 4). The overall inappropriateness of antibiotic use was 28.3% (Figure 2).

**Figure 3:**
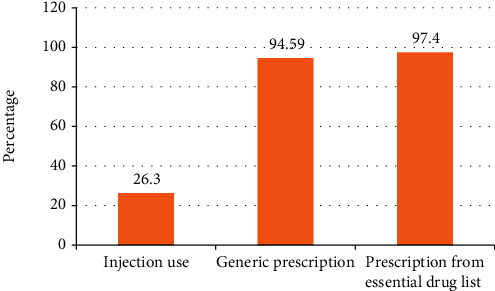
Appropriate drug use based on WHO core indicators.

As indicated in Figure 3, from the total prescriptions, 26.3% of antibiotics were administered by injections. Rational drug use evaluation using WHO core indicators also showed that the proportion of generic prescription was 94.59%. The percentage prescription from the National Essential Drug List was 97.4%.

## 4. Discussion

This study will provide information regarding the status of appropriate antibiotic use for healthcare professionals, policy makers, and healthcare management at different levels. Information obtained from the study may help gearing prescription practices and health care policy improvement on pediatric antibiotic use. It will also serve as a base line data for researchers who are interested to conduct similar studies. A total of 692 pediatric patients' medical charts were reviewed in this study. In our study, the common reasons for antibiotic prescription were sever pneumonia, tonsillitis, cellulitis, and severe acute malnutrition. The results were slightly similar to the study done in pediatric wards of Ayder Referral Hospital, Tigray region, northern Ethiopia, where the most common diagnosis was pneumonia followed by sever acute malnutrition (SAM) [26] and a study from Dessie Referral Hospital, North East Ethiopia, which indicates that the most common diagnosis was sever pneumonia [27]. Other study done in pediatric ward of Jimma University Specialized Hospital also indicates that the most frequent clinical indication for antibiotic prescription was severe pneumonia [1]. This might be due to similar prevalence of infectious diseases.

The findings of the study showed that the most frequently prescribed single antibiotic agent was ceftriaxone followed by cloxacillin and amoxicillin. Studies conducted in pediatric ward of Ayder Referral Hospital, Tigray region, northern Ethiopia [25], and Dessie Referral Hospital, North East Ethiopia [27], also identified a similar result. This might be due to similar medical condition for which antibiotic agents were prescribed. However, the result was different from other study done in pediatric wards of Nigeria which showed that crystallin pencillin was the most frequently prescribed antibiotic [28]. Another study done in pediatric hospital of Erode, Tamilandu, India, also indicated that the most frequently prescribed individual antimicrobial drug was cefotaxime [29].

Regarding combination antibiotics use, the most frequently prescribed antibiotic combination was ceftriaxone with amoxicillin. Ampicillin with gentamicin and ceftriaxone with metronidazole were the second and third most prescribed antibiotic combinations, respectively. This was different from study done in pediatric ward of Hawassa University referral hospital, Ethiopia, which showed that combination of ampicillin with gentamicin took the largest portion, followed by chloramphenicol with cloxacillin [30]. A study done in another part of Ethiopia, Dessie Referral Hospital revealed that the most commonly prescribed multiple drugs were ampicillin with gentamicin. In this study, cloxacillin with chloramphenicol and ceftriaxone with gentamicin were the other most prescribed combination antibiotics [27].

The majority (94.59) of the drugs were prescribed by generic name, and the rest were prescribed by their brand names which was not in line with WHO guidelines recommendation of 100% generic prescription. The proportion of generic prescription was lower compared with findings by [31] results of a study in Saudi Arabia (38.06%) [5].

Percentage of encounters with injections (26.3) was relatively higher compared to WHO standards (13.4-24.1) [22]. This is higher than a report from University of Gondar Comprehensive and Specialized Hospital Outpatient Pharmacy which indicated that the proportion of drugs given by injections was 6.3% [25]. The difference could be due to a focus of the study to outpatient pharmacy, while the result of our study represents inpatient and outpatient pediatric wards. In addition, our study was limited to antibiotic use which may also contribute for differences from other studies, despite a 100% recommendation by WHO prescription by generic name and from National Essential Drug List that were not in accordance with the standards [22, 23].

In our study, 28.3% of antibiotic prescriptions were inappropriate. This result was higher compared to a study conducted in Shenen Gibe Hospital which showed that 33 (10.1%) antibiotics were prescribed inappropriately [19]. The overall prevalence was a bit lower than result from Dessie Referral Hospital, Northeast Ethiopia (58.07). This difference might be due to the professional knowledge, skills, integrity to their works, and also unavailability of first line drugs. Another study conducted at Nekemte Referral Hospital, East Wollega Zone, Oromia Region, West Ethiopia, also showed that 11.80%, 2.95%, and 23.06% were not appropriate dose, frequency, and duration, respectively [16].

As indicated in Table 5, children with 6 to 10 years (AOR = 3.225; CI = 1.080 − 9.630; *P* = .036) and 11-18 years (AOR = 18.691; CI = 5.156 − 67.756; *P* = .000) had the highest odds of inappropriate drug therapy. This might be due to prescribers' perception of considering school age and adolescents as adults; they might tend to give adult doses without considering their age and weight-based doses. Regarding drug classes, cephalosporin use was characterized by higher odd of inappropriate use (AOR = 1.990; CI = .905 − 4.379; *P* = .087). A reason for high dose, high frequency, and prolonged duration prescription of cephalosporins and penicillins may be due to their wider therapeutic indices [32].

## 5. Strength of the Study

To the best of its strength, our study was done on inpatient and outpatient pediatric wards; we used large sample size, and a five year data was evaluated to assess antibiotic prescribing pattern and appropriateness of use. Appropriateness was assessed by using two guidelines (WHO and STG). In addition, the association between predictor and outcome variables was measured.

## 6. Limitation of the Study

The assessment of core prescribing indicators was limited to antibiotic use. This may not show the actual practices in the pediatric population at the study setting. Besides, a retrospective nature of the study may affect the accuracy of results and cause difficulty to find out more predictor variables. Since the study focus on a single facility, results may not be generalized to all hospitals. To address the limitations, we recommend further multicentered and prospective studies.

## 7. Conclusion

This study showed that ceftriaxone and ceftriaxone+amoxicillin were the most frequently used single and combination antibiotics, respectively. Our study revealed 28.3% of inappropriate antibiotic use at Adigrat General Hospital described by irrational indication, dosing, frequency, and duration. Advanced age of children was independent determinant of inappropriate antibiotic prescription in pediatric patients.

## 8. Recommendations

Rational use of drugs could be fostered through an immense work to access standard treatment guidelines; involvement of drug and therapeutic committee; provision of problem-based training in pharmacotherapy; continuing education; through ensuring availability, accessibility, and affordability of drugs; and enhancing the capacity of drug information centers. The collaborative work of clinical pharmacists should also be considered to promote the endeavors of rational drug use in the pediatric population.

We recommend solidification, proper implementation, and ensuring access to activities proved to be useful and effective in promoting appropriate drug use. The results of this study can be used by concerned stakeholders, researchers, and policymakers to improve prescribing practice across hospitals.

## Figures and Tables

**Table 1:** Social demographic characteristics of pediatric patients at the Adigrat General Hospital pediatric ward from December 1, 2018 to April 30, 2019 (*n* = 692).

Social demographic characteristics		Frequency	%
Age	< 1 month (neonates)	62	8.96
	1month- 1years (infant)	66	9.56
	>1-5 years	288	41.62
	>5-10 years	182	26.30
	>10-18 years	94	13.53
Sex	Male	350	50.6
	Female	342	49.4
Duration of hospitalization	1-4 days	262	37.86
	5-9 days	340	49.13
	10-14 days	56	8.09
	15-19 days	32	4.62
	>20 days	2	0.29

**Table 2:** Commonly prescribed single antibiotics in pediatric patients at Adigrat General Hospital December 1, 2018 and April 30, 2019 (*n* = 320).

Antibiotics agents	Frequency	Percentage (%)
Ceftriaxone	147	45.91
Cloxacillin	45	14.17
Amoxicillin	41	12.81
Ciprofloxacin	24	7.36
Ampicillin	22	6.81
Cotrimoxazole	20	6.13
Chloramphenicol	11	3.41
Clarithromycin	7	2.04
Metronidazole	4	1.36
Total	320	100.00

**Table 3:** Distribution of combination antibiotic therapy in pediatric patients at Adigrat General Hospital (*n* = 718).

Antibiotics combinations	Frequency	%
Ceftriaxone+amoxicillin	200	27.86
Ampicillin+gentamicin	195	27.16
Ceftriaxone +metronidazole	70	9.75
Metronidazole+cloxacillin	52	7.24
Ceftriaxone+ cloxacillin+metronidazole	31	4.32
Ceftriaxone+augmentin	40	5.57
Ceftriaxone + gentamicin	35	4.87
Ceftriaxone+ciprofloxacin	30	4.18
Ceftriaxone+cephalexin	10	1.39
Cloxacillin +gentamicin	20	2.79
Ceftriaxone+CAF	15	2.09
Ceftriaxone+cloxacillin	10	1.39
Ampicillin+cipirofloxacillin	10	1.39
Total	718	100.00

**Table 4:** Appropriateness of dosage regimen of overall antibiotics prescribed at pediatric ward of Adigrat General Hospital December 1, 2018 and April 30, 2019 (*n* = 692).

Parameters	Antibiotic regimen		Number	Percentage
Dose	Appropriate		566	81.79
	Inappropriate	Under dose	100	14.45
		Over dose	26	3.76
Frequency	Appropriate		676	97.69
	Inappropriate	Less frequent	16	2.31
		
Duration	Appropriate		622	89.88
	Inappropriate		70	10.12
Treatment	Appropriate		670	96.82
	Unnecessary antibiotic		22	3.18

**Table 5:** Factors associated with inappropriate use of antibiotics using multivariate logistic regression, Adigrat General Hospital, 2019 (*n* = 692).

	Variable	Appropriateness		AOR (ci)	*P* value
		Yes	No		
Gender	Male	260 (74.29)	90 (25.71)	—	—
	Female	236 (69.01)	106 (30.99)	.916 (.375-2.235)	.846
Age	<1 month	52 (83.87)	10 (16.13)	—	—
	1 month-1 year	54 (82.87)	12 (17.13)	1.317 (.366-4.736)	.673
	1 year-5 years	222 (77.15)	66 (22.85)	1.621 (.564-4.6620	.370
	6 years-10 years	122 (67.20)	60 (32.80)	3.225 (1.080-9.630)	.036
	11 years-18 years	44 (47.00)	50 (53.00)	18.69 (5.156-67.756)	.000
Drugs	Penicillins	190 (72.52)	72 (27.48)	.946 (.427-2.094)	.891
	Cephalosporins	142 (64.55)	78 (35.45)	1.990 (.905-4.379)	.087
	Fluoroquinolones	22 (64.71)	12 (35.29)	1.245 (.342-4.528)	.739
	Aminoglycosides	68 (75.56)	22 (24.44)	.843 (.127-3.34)	.467
	Macrolides	40 (76.92)	12 (23.08)	—	—

## Data Availability

The datasets generated and/or analyzed during the current study are available on the corresponding author and will be submitted upon request for securing confidentiality.

## References

[1] Shivaleela J. K., Revankar S., Vedavati H., Chidanand K. N., Jean L. M. (2014). A study of prescription pattern of antibiotics in pediatric in-patients of Mc-Gann Teaching Hospital Shivamogga Institute of Medical Sciences (SIMS), Shivamogga, Karnataka. *IOSR Journal of Dental and Medical Science*.

[2] Rankine-Mullings A. E., Owusu-Ofori S. (2017). Prophylactic antibiotics for preventing pneumococcal infection in children with sickle cell disease. *Cochrane Database of Systematic Reviews*.

[3] Solano-Barquero M., Montero-Salguero A., León-Alán D., Santamaría-Ulloa C., Mora A. M., Reyes-Lizano L. (2018). Prevalence of parasitosis in children aged 1 to 7 years in vulnerable condition in the south central region of Costa Rica. *Acta Médica Costarricense*.

[4] Woldu M. A., Suleman S., Workneh N., Berhane H. (2013). Retrospective study of the pattern of antibiotic use in Hawassa University referral hospital pediatric ward, southern Ethiopia. *Journal of Applied Pharmaceutical Science*.

[5] Alakhali K. M. (2014). prescribing pattern of antibiotics in pediatric patients in the Jazan region, Kingdom of Saudi Arabia. *RGUHS Journal of Pharmaceutical sciences*.

[6] Maheshwari P., Ravichandiran V., Hemanth Bhaskar K. K., Vydehi S. S. K., Baig T. S., Shahel S. N. (2015). Prescribing patterns of antibiotics in paediatrics for respiratory tract infections/disorders in tertiary care hospital. *Asian Journal of Pharmaceutical and Clinical Research*.

[7] Matera M. G., Rogliani P., Ora J., Cazzola M. (2018). Current pharmacotherapeutic options for pediatric lower respiratory tract infections with a focus on antimicrobial agents. *Expert opinion on pharmacotherapy*.

[8] Thapaliya K., Shrestha S., Bhattarai S., Basnet D., Chaudhary R. K. (2015). Prescribing pattern of antibiotics in pediatric hospital in Chitwan district in Nepal. *World Journal of Pharmaceutical Sciences*.

[9] Ladak S. S., Chan V. W., Easty T., Chagpar A. (2007). Right medication, right dose, right patient, right time, and right route: how do we select the right patient-controlled analgesia (PCA) device?. *Pain Management Nursing*.

[10] Uppal R., Chhabra A., Narang A. (1998). Pattern of drug use in neonatal intensive care unit. *Indian pediatrics*.

[11] Joda A. E., Aderemi-Williams R. I. (2013). A comparative study of prescribing patterns in two tertiary care teaching hospitals in Lagos, Nigeria. *IJPP*.

[12] Vieira P. N., Vieira S. L. (2017). Irrational use of antimicrobials and resistance in hospitals. *Arquivos de Ciências da Saúde da UNIPAR*.

[13] Nambiar R., Shah D., Ajbani K. (2018). Evaluation of pyrosequencing for extensive drug resistance-defining anti-tuberculosis drugs for use in public healthcare. *Tuberculosis*.

[14] Xie X., Li X., Sheng Y., Gu B. (2019). *Prediction of Antimicrobial Resistance Based on Random Forest Algorithms*.

[15] Asefa L., Bayissa G., Abera Z. (2016). Antibiotics use evaluation for pediatrics at Nekemte Referral Hospital, East Wollega Zone, Oromia region, West Ethiopia. *World Journal of Medical Sciences*.

[16] Getachew E., Aragaw S., Adissie W., Agalu A. (2013). Antibiotic prescribing pattern in a referral hospital in Ethiopia. *African Journal of Pharmacy and Pharmacology*.

[17] Zeleke A., Chanie T., Woldie M. (2014). Medication prescribing errors and associated factors at the pediatric wards of Dessie Referral Hospital, Northeast Ethiopia. *International archives of medicine*.

[18] Kebede H. K., Gesesew H. A., Woldehaimanot T. E., Goro K. K. (2017). Antimicrobial use in paediatric patients in a teaching hospital in Ethiopia. *PLoS One*.

[19] Barghouthi Achalu T., Mensa M. (2017). Retrospective drug use pattern of antibiotics in pediatric ward of Shenan Gibe hospital, Oromia Region, Ethiopia. *Journal Antibiotic Research*.

[20] https://www.who.int/maternal_child_adolescent/documents/child_hospital_care/en/. Accessed: November 25, 2018

[21] http://www.fmhaca.gov.et/wp-content/uploads/2014/03/STG-General-Hospital.pdf. Accessed November 26, 2018

[22] World Health Organization (1993). *How to investigate drug use in health facilities: selected drug use indicators*.

[23] Food, medicine and healthcare administration and control authority of Ethiopia *National Essential Medicine List Fifth Edition*.

[24] Yimenu D. K., Emam A., Elemineh E., Atalay W. (2019). Assessment of antibiotic prescribing patterns at outpatient pharmacy using World Health Organization prescribing indicators. *Journal of Primary Care & Community Health*.

[25] Gebretsadik Z., Gebrehans M., Getnet D., Gebrie D., Alema T., Belay Y. B. (2017). Assessment of drug-drug interaction in Ayder Comprehensive Specialized Hospital, Mekelle, Northern Ethiopia: a retrospective study. *BioMed Research International*.

[26] Mezgebe H. B., Tadesse B., Legesse B. (2015). Antibiotics prescribing pattern in pediatric unit of Ayder referral hospital, Tigray region, Northern Ethiopia. *JSIR*.

[27] Alemnew G., Atnafie S. A. (2015). Assessment of the pattern of antibiotics use in pediatrics ward of Dessie Referral Hospital, North East Ethiopia. *International journal of medicine and medical sciences*.

[28] Arute J. E., Adigom D. O., Erah P. O., Eichie F. E., Eniojukan J. F. (2011). Antibiotic prescription pattern in the paeditric ward of a tertiary health-care facility in southern Nigeria. *Journal of Pharmaceutical and allied sciences*.

[29] Shamshy K., Begum I. M., Perumal P. (2011). Drug utilization of antimicrobial drug in pediatrics population in a tertiary care hospital in Erode, Tamilnadu, India. *International Journal of PharmTech Research*.

[30] Minyahil A., Woldu S. S. (2013). Netsanet Workneh and Hafty barhane. Retrospective study of the pattern of antibiotic use in hawassa University Referral Hospital Pediartric ward, southern Ethiopia. *Journal of Applied Pharmaceutical Science*.

[31] Shrestha B., Dixit S. M. (2018). The Assessment of drug use pattern using WHO prescribing indicators. *Journal of Nepal Health Research Council*.

[32] Floege J., Johnson R. J., Feehally J. (2010). *Comprehensive Clinical Nephrology E-Book*.

